# Novel developments in the study of estrogen in the pathogenesis and therapeutic intervention of lymphangioleiomyomatosis

**DOI:** 10.1186/s13023-024-03239-1

**Published:** 2024-06-14

**Authors:** Jingye Tai, Shihua Liu, Xinping Yan, Luantai Huang, Yingxin Pan, Hongyuan Huang, Zhen Zhao, Beini Xu, Jie Liu

**Affiliations:** 1 Nanshan College of Guangzhou Medical University, Guangzhou, 510515 China; 2 The First Affiliated Hospital of Guangzhou Medical University, Guangzhou, 510120 China; 3 The First Clinical College of Guangzhou Medical University, Guangzhou, 510515 China; 4 Department of Pulmonary and Critical Care Medicine, The First Affiliated Hospital of Guangzhou Medical University, Guangzhou, 510120 China; 5 National Center for Respiratory Medicine, Guangzhou, China; 6 Guangzhou Institute for Respiratory Health, Guangzhou, China; 7 State Key Laboratory of Respiratory Diseases, Guangzhou, China; 8 National Clinical Research Center for Respiratory Disease, Guangzhou, China

**Keywords:** LAM, Estrogen, Pregnancy, Estrogen receptor inhibitor

## Abstract

**Objective:**

This study aimed to enhance the understanding of the role of estrogen in lymphangioleiomyomatosis(LAM) and to conclude the impact of estrogen-altering events on the condition and recent advances in estrogen-based treatments for LAM.

**Results:**

LAM development is strongly linked to mutations in the tuberous sclerosis gene (*TSC1/2*) and the presence of estrogen. Estrogen plays a significant role in the spread of TSC2-deficient uterine leiomyoma cells to the lungs and the production of pulmonary LAM. Menstruation, pregnancy, estrogen medication, and other events that cause an increase in estrogen levels can trigger the disorder, leading to a sudden worsening of symptoms. Current findings do not support using estrogen-blocking therapy regimens. However, Faslodex, which is an estrogen receptor antagonist, presents new possibilities for future therapeutic approaches in LAM.

**Conclusion:**

Estrogen is crucial in the development and spread of LAM. The use of estrogen inhibitors or estrogen receptor antagonists alone does not provide good control of the disease or even poses a greater risk, and the use of a combination of mTOR receptor inhibitors, complete estrogen receptor antagonists, estrogen inhibitors, and autophagy inhibitors targeting important signaling pathways in LAM pathogenesis may be of greater benefit to the patient.

## Background

Lymphangioleiomyomatosis (LAM) is a rare disease that mainly occurs in women of childbearing age, may also occur in postmenopausal women, and is extremely rare in men. There are two distinct clinical types of LAM: sporadic LAM (S-LAM) and tuberous sclerosis (TSC)-associated LAM (TSC-LAM), which is related to the TSC complex. Moreover, clinical data suggest that menstruation, pregnancy, and estrogen medication worsen the symptoms in certain individuals. Furthermore, estrogen significantly impacts the multiplication of LAM tumor cells, and it also expedites the spread of TSC2 mutant tumor cells and stimulates LAM cell expansion. Multiple clinical trials have investigated the efficacy of estrogen receptor antagonists and antiestrogen medications in treating LAM. This article provides a concise overview of the advancements made in understanding the role of estrogen in LAM development and treatment. To offer a valuable resource for clinicians and researchers is the primary objective.

## The role of estrogen in LAM pathogenesis

Recent studies have shown that mutations in the *TSC1/2* genes are closely related to LAM pathogenesis. The *TSC1/2* genes produce hamartin and tuberin, which both engage with other proteins to create a tumor suppressor complex and interact with mammalian target of rapamycin (mTOR) complex 1(mTORC1). The TSC1–TSC2 protein complex inhibits mTORC1 via the small GTPase Rheb, a direct target of the GTPase-activating structural domain of the TSC2 protein potato block protein [1]. 4E-binding protein(4EBP) phosphorylation by mTORC1 promotes its release from eukaryotic translation initiation factor 4E(eIF4E) and activation of ribosomal S6 kinase 1/2 (S6K1/2) leads to subsequent phosphorylation downstream. This is followed by the inactivation of eukaryotic elongation factor 2 kinase (eEF2K) and eukaryotic elongation factor 2 (eEF2) [2]. The primary function of mTORC1 is to regulate and inhibit tumor development. LAM is characterized by a pathological mutation in the *TSC1/2* gene, which hinders hamartin and tuberin production. This disruption leads to mTORC1 hyperactivation, thereby triggering an excessive synthesis of proteins and uncontrolled cell growth, ultimately ending in tumor development.

Individuals with TSC exhibit a balanced distribution of men and women. However, among patients with TSC-LAM and S-LAM, most cases include women of childbearing age. Immunohistochemical analysis of tissue samples from patients with LAM frequently reveals the presence of estrogen and progesterone receptors in lung lesions. There is a prevailing concept suggesting that LAM cells can originate from the uterus [3]. It is hypothesized that a correlation exists between estrogen and LAM, and the exact mechanism remains unclear [4].

**Fig. 1:**
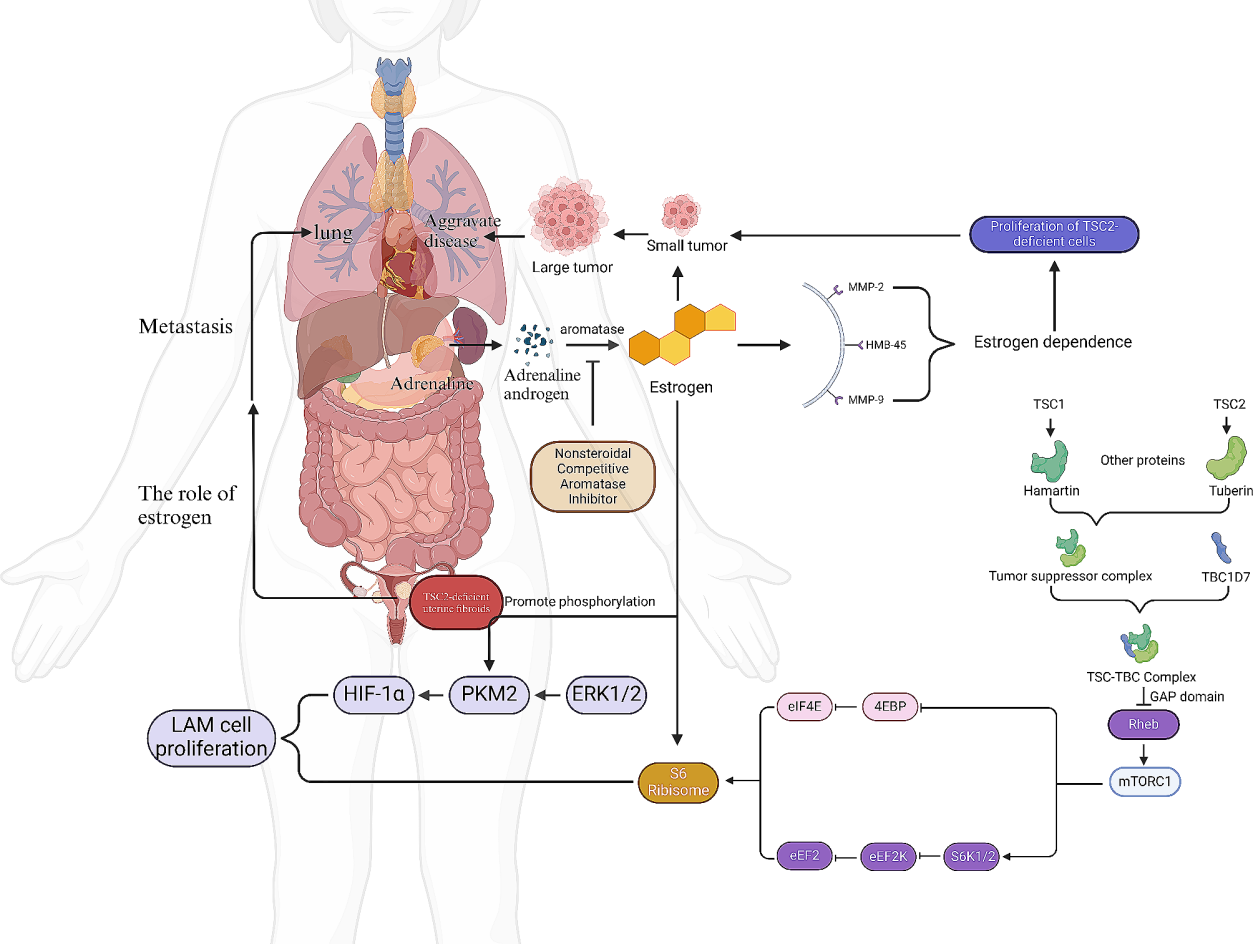
Diagram of the mechanism of action of estrogen in lymphangioleiomyomatosis (LAM) MMP2: matrix metalloproteinase 2; MMP9: matrix metalloproteinase 9; NE: neutrophil elastase; HMB-45: melanoma-associated antigens; Hamartin: hamartoma protein; Tuberin: potato globulin

Molecularly, extracellular signal-regulated kinase 1/2(ERK1/2) activation plays a significant role in endothelial cell proliferation, migration, and survival. Within cells lacking TSC2, ERK1/2 activation results in pyruvate kinase M2(PKM2) phosphorylation at Ser37. This phosphorylation event influences Hypoxia-inducible factor 1-alpha(HIF-1α) transcription, which subsequently promotes cell proliferation. Notably, PKM2 phosphorylation is reliant on estrogen, highlighting the significant role of estrogen in activating the ERK pathway. Estrogen increases PKM2/Ser37 phosphorylation and induces nuclear translocation of the phosphorylated PKM2. These effects were reversed with the estrogen receptor antagonist Faslodex [5]. Simultaneously, estradiol (E2) stimulates a robust and biphasic activation of ERK2 and transcription of the late response-gene *Fra1* associated with epithelial-to-mesenchymal transition [2]. Research has demonstrated that the removal of estrogen not only suppresses the production of the abovementioned proteins but also suppresses S6K production. Furthermore, its inhibitory impact on S6K is more significant than that of TSC2 [6]. Matrix metalloproteinases (MMPs) 2 and 9, neutrophil elastase, and HMB-45 are substances that rely on estrogen and stimulate the growth of LAM cells when TSC is not present (Fig. 1) [7]. Furthermore, regarding the glucose metabolism pathway, E2 reactivates Akt-induced membrane translocation of glucose transport proteins (GLUT1 or GLUT4) in TSC2-deficient cells in vivo and in vitro and increases glucose uptake in an Akt-dependent manner. Increases cellular NADPH levels, decreases reactive oxygen species levels, and enhances cell survival in response to oxidative stress [8].

Preclinical investigations have shown that estrogen stimulates the growth of TSC2 mutant cells and LAM cells. A strong positive feedback loop exists between E2 and tumor factors, which induces neutrophil expansion, subsequently exacerbating tumor growth and neutrophil-stimulating factor production, thereby leading to sustained tumor growth in TSC2-deficient tumors [9]. This growth is linked to the spread of TSC2-deficient uterine leiomyoma cells to the lungs and the development of pulmonary LAM. Postmenopausal women rely on the aromatase enzyme to convert adrenal androgens to estrogens in the periphery, which is the primary source of estrogen. By using letrozole, a nonsteroidal competitive aromatase inhibitor, estrone and E2 levels in the bloodstream are significantly reduced. This reduction helps alleviate the effects of LAM without interfering with adrenocorticotropic hormone, aldosterone, or thyroid hormone synthesis [10].

## Impact of estrogen-altering events on LAM

### Effects of pregnancy events on LAM

LAM may be exacerbated by elevated estrogen levels during pregnancy. Mitchell [11] examined 145 individuals with TSC-LAM and reported that the occurrence of renal problems (57% vs. 67%; *P* = 0.62) and pneumothorax complications (40% vs. 38%; *P* = 1.00) were not statistically significant between the pregnant and nonpregnant groups. Urban [12] reviewed 69 patients with LAM and observed that 20% had onset during pregnancy and 14% had significantly worse symptoms during pregnancy. Johnson and Tattersfield [13] examined a group of 50 patients with LAM, 28 of whom had been pregnant and 27 of whom had given birth. These patients experienced a greater number of complications during pregnancy than when they were not pregnant. Additionally, seven patients developed the primary symptom of LAM either during or after pregnancy, with four experiencing it during pregnancy and three after pregnancy. Cohen MM [14] investigated 328 women with LAM and showed that patients diagnosed with LAM during pregnancy had a higher incidence of pneumothorax (67%), miscarriage (7%), and preterm labor (47%). Decreased lung function during pregnancy is common, and results from an analysis including 89 patients with LAM [15] showed that the group with decreased lung function had a significantly higher frequency of pregnancy than the nondecreased group. Munshi A surveyed 38 (76%) of 50 respondents with at least one pregnancy, for a total of 80 pregnancies, and observed that 34% (13/38) of the patients were diagnosed with LAM due to respiratory symptoms during pregnancy, with spontaneous pneumothorax (SP) being the main manifestation in most patients (10/13, 77%). Of the 38 pregnant patients, 11 (29%) experienced SP at least once during pregnancy [16]. Current research findings showed that a significant number of patients are diagnosed with LAM during pregnancy, and the condition worsens during pregnancy and menstruation, suggesting that pregnancy events may accelerate the natural course of LAM.

A study examining pregnancy and LAM [14] noted that patients who were diagnosed with LAM during pregnancy experienced poorer pregnancy outcomes than those detected before or after pregnancy. Specifically, the group diagnosed during pregnancy showed significantly higher rates of preterm deliveries and miscarriages (53%, *n* = 8) than those diagnosed before (20%, *n* = 3) or after pregnancy (15%, *n* = 50). A study of 230 patients with LAM [17] reported that two-thirds had a pregnancy wherein 66.9% had successful deliveries, whereas 16.7%, 15.0%, and 1.4% had spontaneous, therapeutic, and stillbirths, respectively; approximately 25% of the patients with LAM had respiratory symptoms that worsened during pregnancy. A study [14] showed that LAM has an impact on fertility, and 37% of the 328 women with LAM had no history of childbearing compared with 15% in the general population. Most women refused to have children for the following reasons: (1) fear that pregnancy will worsen LAM, (2) fear of passing on TSC to offspring, and (3) advice from healthcare professionals.

The European Respiratory Society (ERS) Guidelines for the Diagnosis and Management of LAM emphasize that [18] patients with LAM are recommended to be cautious about pregnancy, considering the possible involvement of estrogens in the pathogenesis of LAM and the risk of disease progression and abnormal pregnancy outcomes. Risks include the following: (1) pneumothorax, celiac disease, and AML bleeding; (2) disease progression during pregnancy; and (3) severe dyspnea or hypoxemia during low baseline lung function or complications. As the population with LAM comprises women of childbearing age, a high demand for pregnancy is observed; in the current study, patients with mild or moderate lung function decline were relatively safe to become pregnant with assessment of HRCT grading, lung function status, and other comorbidities. Shen L et al. mentioned the following seven recommendations for pregnancy evaluation in patients with LAM: (1) degree of LAM on high-resolution computed tomography, (2) baseline lung function and rate of lung function decline, (3) previous history of pneumothorax or celiac disease, (4) presence and size of renal AML or retroperitoneal lymphangio-smooth muscle tumor, (5) TSC, (6) sirolimus therapy, and (7) previous pregnancy history [19].

### Contraceptives

Contraceptives usually contain estrogen, and elevated estrogen levels may exacerbate LAM. The results of a survey of 39 patients with LAM [20], 25 (65%) of whom were confirmed as current or past users of estrogen-containing contraceptives, showed a correlation between the use of this class of contraceptives and LAM (preponderance ratio, 6.500; 95% confidence interval, 1.199–35.230). A study of 91 patients with LAM [21] reported that the rate of oral contraceptive use was approximately 27%, similar to the rate in the general population. A significant difference was observed between the age at which LAM-related symptoms developed in patients using oral contraceptives versus those not using oral contraceptives (29.2 ± 4.7 vs. 32.9 ± 8.0; *p* = 0.0397), suggesting that oral contraceptives can promote early LAM onset. The ERS Guidelines for the Diagnosis and Management of Lymphatic Vascular Smooth Myelomatosis recommend that women with LAM should avoid estrogen-containing treatments. Some studies have reported that hormone products are absolutely contraindicated for LAM. Exogenous estrogen administration can worsen lung disease. Although progesterone is one of the classic treatments for this disease, no studies on progestin-only contraceptives have been conducted. In general, barrier methods of contraception or intrauterine devices(IUDs) should be recommended for these women [22].

### Other estrogen-related events

Moreover, the decline in postmenopausal estrogen levels can affect patients with LAM, and a cohort analysis study [23] showed that menopausal status significantly influenced LAM progression. In the placebo group, pre-menopausal patients declined 5-fold faster than post-menopausal patients (mean ± SE FEV1 slope, − 17 ± 3 vs. −3 ± 3 mL·month − 1; *p* = 0.003). Upon sirolimus treatment, both the premenopausal (− 17 ± 3 vs. −1 ± 2 mL·month − 1; *p* < 0.0001) and postmenopausal patients (− 3 ± 3 vs. 6 ± 3 mL·month − 1; *p* = 0.04) exhibited a beneficial response in mean ± SE FEV1 slope compared with the placebo group. Another study [24] demonstrated that age and menopausal status were significant determinants of SP risk in patients with LAM. The median age at SP onset was 36 (29–43) years in a cohort, wherein 65 postmenopausal patients with LAM remained free from SP and 9 (13.8%) developed SP after menopause (*p* < 0.001). Similarly, in another cohort, the median age at SP onset was 31 (25–38) years, wherein 13 postmenopausal patients with LAM had not experienced SP and 2 (15%) developed SP after menopause (*p* = 0.008). These findings suggest that decreased estrogen levels following menopause can reduce SP risk in individuals with LAM.

In a case report [25], two women diagnosed with LAM presented with abdominal lymphangioleiomyomas that exhibited growth patterns influenced by changes in estrogen levels during the menstrual cycle. As abnormal smooth muscle cells express both estrogen and progesterone receptors but are particularly responsive to altered estrogen levels, elevated progesterone levels following ovulation also contribute to delaying the growth of LAM lesions. Furthermore, estrogen level fluctuations throughout the menstrual cycle can impact LAM progression.

## Studies related to estrogen therapy for LAM

### Antiestrogen therapy

Currently, antiestrogen therapy is only used clinically in over- adaptation patients with LAM and has not been recognized in clinical trials. In a case report [26], two of three patients who underwent oophorectomy showed improvement, and one patient had stable disease without deterioration. One patient in another case report [27] also improved following ovariectomy. There are also several case reports of progesterone therapy [28–32]. In a controlled observational study of 275 patients with LAM [33], progesterone-treated patients showed a higher rate of diffusing capacity for carbon monoxide(DLCO) decline than nonprogesterone-treated patients, and no significant difference in the annual rate of decline in FEV1 was noted. In a case study of nine patients treated with the gonadotrophin-releasing hormone(GnRH) agonist goserelin [34], the mean increases in FEV1 and FVC were 80 and 130 mL, respectively. Another case study of 11 patients treated with the GnRH agonist treprostinil [35] revealed the following results: positive treatment outcomes were not observed, the pulmonary function of the participants was significantly decreased in all of them, and the treprostinil treatment may result in bone mineral density decrease. Evidently, antiestrogen therapy for LAM does not provide positive effects for patients and even poses greater risks. As a result of the latest research and applications in clinical practice, the recommendations do not support hormone therapy as a LAM treatment [36].

### Estrogen receptor antagonist therapy

**Fig. 2:**
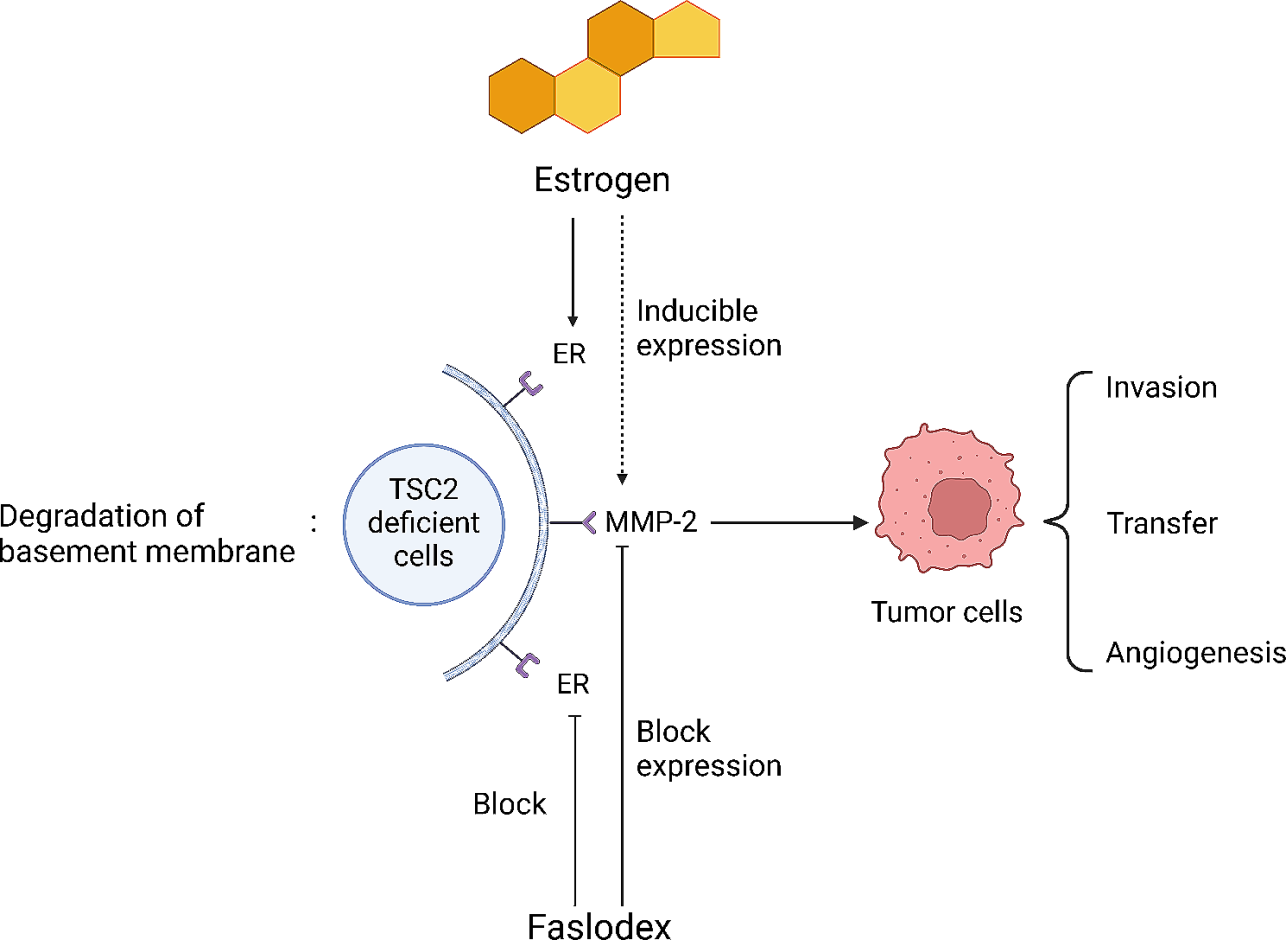
Faslodex mechanism of action ER: estrogen receptors; MMP2: matrix metalloproteinase 2; Faslodex is an estrogen receptor antagonist that inhibits the expression of estradiol-enhanced extracellular matrix (ECM) genes and reduces MMP2 expression and activity. However, it does not affect estradiol’s promotion of primary tumor growth in TSC2-deficient cells. Additionally, Faslodex does not participate in other estrogen agonist activities

We have now identified inactivating mutations in two alleles of the *TSC1* or *TSC2* genes in LAM cells from patients with TSC-LAM and S-LAM [37, 38]. Renal angiomyolipomas affect approximately 60% of women with S-LAM, and renal angiomyolipoma cells from women with S-LAM also have *TSC2* mutations, supporting the theory that LAM cells can metastasize to the lungs [39]. Recent research showed that esteriol promotes circulatory tumor cells and pulmonary transplants of tuberculosis-deficient cells by increasing MMP2 production and activity of TSC2 faulty cells [4, 40]. Faslodex, an estrogen receptor antagonist, was used in a study [3] wherein mice were implanted with either placebo or E2 1 week before cell inoculation and subsequently treated with fulvestrant (1 mg/day intramuscular injection). At 8 weeks following inoculation, the mean tumor area in E2-treated mice was 251 ± 65 mm² compared with 107 ± 47 mm² (*P* < 0.05) in placebo-treated mice. Faslodex had no effect on E2 promotion of primary tumor growth in TSC2-deficient cells. Furthermore, Faslodex inhibited E2-enhanced ECM gene expression and decreased MMP2 expression and activity. In a study on E2- and tamoxifen-treated cells, E2 stimulated cell growth with p44/42 mitogen-activated protein kinase (MAPK) phosphorylation at 5 min and increased c-myc expression at 4 h. Moreover, tamoxifen citrate stimulated cell growth and was associated with increased p44/42 MAPK phosphorylation and c-myc expression, suggesting that tamoxifen has an agonistic effect on vascular smooth muscle lipoma cells [41]. In contrast to tamoxifen and other selective ER modulators, Faslodex does not affect other estrogen agonist activities, whereas tamoxifen stimulates angiomyolipoma cell growth in patients with LAM. Therefore, complete estrogen receptor antagonists (Faslodex) may offer greater benefits to patients than selective estrogen receptor modulators (tamsulosin). However, no extensive clinical trials to demonstrate the safety and efficacy of estrogen receptor antagonists for the treatment of lung metastases in patients with LAM have been conducted. However, it provides a novel clinical idea to use Faslodex for treating TSC2 mutant LAM (Fig. 2).

## Summary

Estrogen plays a significant role in the pathogenesis of LAM, affecting MMP levels in several ways, promoting the metastasis of TSC2-deficient cells, and accelerating LAM cell proliferation. Furthermore, E2 affects the glucose metabolic pathways, increases NADPH levels, and improves cell survival under oxidative stress. Changes in hormone levels under physiological conditions including pregnancy and menstrual events also affect the symptoms of patients with LAM. The positive feedback loop between E2 and tumor factors expands tract neutrophils and exacerbates tumor growth. Therefore, estrogen suppression therapy and estrogen receptor antagonism therapy have become a new research direction; however, based on the results of most of the estrogen-specific therapies available, estrogen suppression therapy is ineffective in controlling LAM progression and even poses a greater risk. Although antiestrogen therapy is not recommended, a large number of clinical trials are needed to determine whether the therapeutic idea of estrogen receptor antagonists can reduce the risk of lung tumor metastasis in TSC2 mutant LAM. The use of estrogen inhibitors or estrogen receptor antagonists alone does not provide good control of the disease or even poses a greater risk, and the use of a combination of mTOR receptor inhibitors, complete estrogen receptor antagonists, estrogen inhibitors, and autophagy inhibitors targeting important signaling pathways in LAM pathogenesis may be of greater benefit to the patient.

## Data Availability

Not applicable.

## Abbreviations

4EBP: 4E-binding protein
DLCO: Diffusing capacity for carbon monoxide
E2: Estradiol
eEF2: Eukaryotic elongation factor 2
eEF2K: Eukaryotic elongation factor 2 kinase
eIF4E: Eukaryotic translation initiation factor 4E
ERK1/2: Extracellular signal-regulated kinase 1/2
FEV1: Forced expiratory volume in 1 s
FVC: Forced vital capacity
GnRH: Gonadotrophin-releasing hormone
HIF-1α: Hypoxia-inducible factor 1-alpha
IUDs: Intrauterine devices
LAM: Lymphangioleiomyomatosis
MAPK: Mitogen-activated protein kinase
MMPs: Matrix metalloproteinases
mTOR: Mammalian target of rapamycin
PKM2: Pyruvate kinase M2
Rheb: Ras homolog enriched in brain
S-LAM: Sporadic lymphangioleiomyomatosis
S6K1/2: Ribosomal S6 kinase 1/2
SP: Spontaneous pneumothorax
TSC1/2: Tuberous sclerosis gene 1/2
TSC: Tuberous sclerosis complex
